# Optimization of an Information Leaflet to Influence Medication Beliefs in Women With Breast Cancer: A Randomized Factorial Experiment

**DOI:** 10.1093/abm/kaad037

**Published:** 2023-07-26

**Authors:** Sophie M C Green, Louise H Hall, David P French, Nikki Rousseau, Catherine Parbutt, Rebecca Walwyn, Samuel G Smith

**Affiliations:** Leeds Institute of Health Sciences, University of Leeds, Leeds, UK; Leeds Institute of Health Sciences, University of Leeds, Leeds, UK; Manchester Centre for Health Psychology, University of Manchester, Manchester, UK; Leeds Institute of Clinical Trials Research, University of Leeds, Leeds, UK; Medicines Management and Pharmacy Services, Leeds Teaching Hospitals NHS Trust Leeds, Leeds, UK; Leeds Institute of Clinical Trials Research, University of Leeds, Leeds, UK; Leeds Institute of Health Sciences, University of Leeds, Leeds, UK

**Keywords:** Breast cancer, Medication beliefs, Optimization, Factorial, Information leaflet

## Abstract

**Background:**

Adherence to adjuvant endocrine therapy (AET) is low in women with breast cancer. Negative beliefs about the necessity of AET and high concerns are barriers to adherence.

**Purpose:**

To use the multiphase optimization strategy to optimize the content of an information leaflet intervention, to change AET beliefs.

**Methods:**

We conducted an online screening experiment using a 2^5^ factorial design to optimize the leaflet. The leaflet had five components, each with two levels: (i) diagrams about AET mechanisms (on/off); (ii) infographics displaying AET benefits (enhanced/basic); (iii) AET side effects (enhanced/basic); (iv) answers to AET concerns (on/off); (v) breast cancer survivor (patient) input: quotes and photographs (on/off). Healthy adult women (*n* = 1,604), recruited via a market research company, were randomized to 1 of 32 experimental conditions, which determined the levels of components received. Participants completed the Beliefs about Medicines Questionnaire before and after viewing the leaflet.

**Results:**

There was a significant main effect of *patient input* on beliefs about medication (β = 0.063, *p* < .001). There was one significant synergistic two-way interaction between *diagrams* and *benefits* (β = 0.047, *p* = .006), and one antagonistic two-way interaction between *diagrams* and *side effects* (β = −0.029, *p* = .093). There was a synergistic three-way interaction between *diagrams, concerns,* and *patient input* (β = 0.029, *p* = .085), and an antagonistic four-way interaction between *diagrams, benefits, side effects,* and *concerns* (β = −0.038, *p* = .024). In a stepped approach, we screened in four components and screened out the side effects component.

**Conclusions:**

The optimized leaflet did not contain enhanced AET side effect information. Factorial experiments are efficient and effective for refining the content of information leaflet interventions.

## Teaser

Factorial experimental designs are an efficient way to optimize the content of information leaflets. Optimizing an information leaflet to support beliefs about a medication prescribed to women with breast cancer led enhanced information about side-effects to be screened out.

## Introduction

Breast cancer is the most common cause of cancer death in women worldwide [1]. Adjuvant endocrine therapy (AET) is prescribed to women with estrogen receptor-positive (ER+) breast cancer for 5–10 years to prevent recurrence and mortality [2–4]. However, many women do not take AET as prescribed [5–7]. Nonadherence to AET increases the risk of recurrence and reduces survival and quality-adjusted life years [8, 9].

Medication beliefs, in the form of low perceived personal need for AET and high concerns about AET (e.g., burden of side effects), are associated with lower AET adherence [6, 10–16]. The Necessity-Concerns Framework (NCF) suggests women weigh up their personal perceived need for AET, against their concerns in a cost-benefit analysis to decide whether to take AET [17].

An extended version of the self-regulation model of illness suggests illness representations could influence key medication beliefs regarding the necessity or concerns of medication [17, 18]. For example, stronger beliefs that AET can reduce the risk of recurrence (treatment control) have been associated with increased necessity beliefs, and reduced concerns [19]. Similarly, better understanding of how AET works (coherence) has been associated with fewer AET concerns, while attributing more physiological sensations (identity) to AET (e.g., side effects) has been associated with increased AET concerns [19]. It has been hypothesized that necessity and concern beliefs mediate the relationship between illness perceptions (e.g., treatment control, coherence) and medication adherence [18–20]. Therefore, illness representations may be potential intervention targets, which could consequently influence necessity and concern beliefs.

There is little understanding regarding effective strategies to target medication beliefs [21–23]. A randomized controlled trial (RCT) found small to moderate effect sizes on medication beliefs using a three-session cognitive behavioral approach [24]. RCTs involving single intervention and control arms can tell us whether the intervention package as a whole is more effective than a comparator, but they do not provide information on which components are affecting the outcome, or whether any components are interacting. This limits our understanding of how we can effectively target medication beliefs.

Medication beliefs are complex, and therefore a multicomponent intervention may be needed to target all aspects of the construct. The multiphase optimization strategy (MOST) is a framework used to optimize multicomponent interventions [25, 26]. MOST consists of three phases. The first and final phases reflect a classical approach in which an intervention package is prepared, and then evaluated, typically with a parallel groups RCT. MOST advocates for an additional optimization phase between the preparation and evaluation phases. In this optimization phase, highly efficient, fully powered experimental designs are used to estimate the main and interaction effects of intervention components [27]. Optimization trials allow intervention developers to screen out components having a negative or null effect on an outcome, or that are not justified based on resource demands. This has the potential to create more effective, affordable, scalable, and efficient intervention packages [28].

We aimed to prepare and optimize an information leaflet intervention, aiming to increase necessity beliefs and reduce concerns about AET. We had three objectives: (i) to evaluate the main effects of each component of the information leaflet on beliefs about AET, (ii) to estimate interactions between components of the information leaflet on beliefs about AET, and (iii) to establish an optimal combination of information leaflet components with regard to changing beliefs about AET.

## Methods

### Preparation Phase: Information Leaflet Intervention Development

**Fig. 1.**
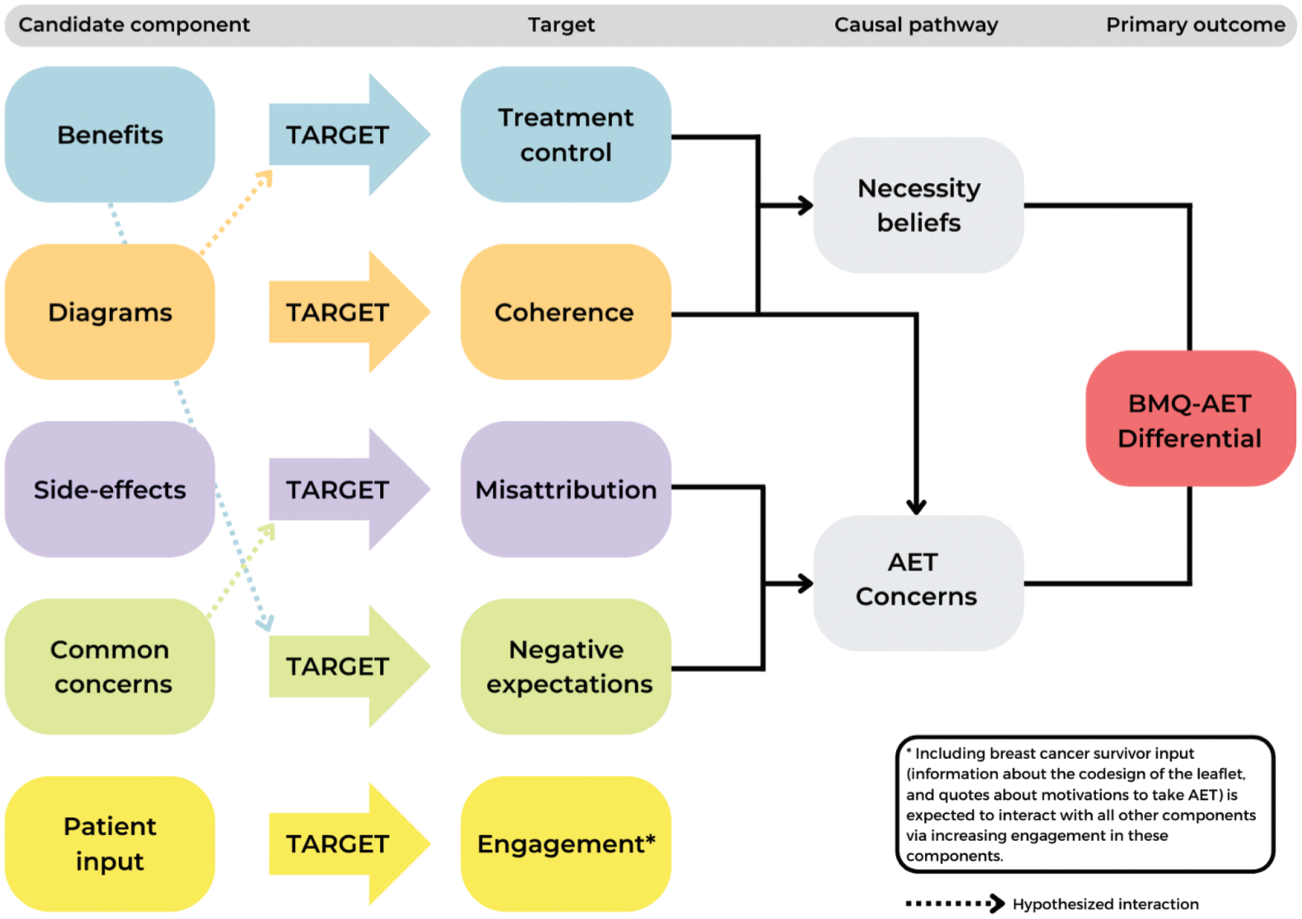
Conceptual model

As part of a wider program of research, we used intervention mapping combined with MOST to develop a written information leaflet to change AET medication beliefs [29]. A written information leaflet was chosen, as it is a low cost, implementable method that can provide accurate information about the benefits and risks of AET, which could encourage more balanced medication beliefs [30–35]. We chose five distinct intervention targets, based on the NCF, self-regulation model, causal learning theory, and existing literature [17, 18, 36]. Our conceptual model details how we hypothesized each component to influence medication beliefs (Fig. 1). The content of the leaflet was developed with our patient group, consisting of five breast cancer survivors with experience taking AET, and a consultant pharmacist with clinical experience of AET. A professional design company designed the leaflet.

### Optimization Phase: Randomized Factorial Screening Experiment

#### Experimental design

We conducted an online, 2^5^ (2 × 2 × 2 × 2 × 2) factorial experiment. The primary outcome was participant’s beliefs about AET. Five candidate components were used as factors with two levels (on vs. off, or enhanced vs. basic). We randomized participants to 1 of 32 experimental conditions, which determined which levels of the components of the information leaflet participants would view (Table 1). Participants could receive any combination of the five components. One author (S.G.) created information leaflet versions corresponding to the experimental condition. A second author (S.S.) reviewed 20% (6 information leaflets) of the intervention information leaflets to check the correct levels of each candidate component were included. The reading level for the 32 versions of the information leaflet ranged from 6.8 to 7.6 on the Flesch–Kincaid reading grade level; between “easy to read” and “fairly easy to read,” respectively [37].

**Table 1.** Experimental conditions in 2^5^ factorial design and number randomized to each condition

	Constant Component	Diagrams	Benefits	Side effects	Common concerns	Patient input	Number randomized
1	Yes	Yes	Enhanced	Enhanced	Yes	Yes	55
2	Yes	Yes	Enhanced	Enhanced	Yes	No	54
3	Yes	Yes	Enhanced	Enhanced	No	Yes	53
4	Yes	Yes	Enhanced	Enhanced	No	No	38
5	Yes	Yes	Enhanced	Basic	Yes	Yes	53
6	Yes	Yes	Enhanced	Basic	Yes	No	56
7	Yes	Yes	Enhanced	Basic	No	Yes	47
8	Yes	Yes	Enhanced	Basic	No	No	58
9	Yes	Yes	Basic	Enhanced	Yes	Yes	45
10	Yes	Yes	Basic	Enhanced	Yes	No	57
11	Yes	Yes	Basic	Enhanced	No	Yes	42
12	Yes	Yes	Basic	Enhanced	No	No	50
13	Yes	Yes	Basic	Basic	Yes	Yes	54
14	Yes	Yes	Basic	Basic	Yes	No	41
15	Yes	Yes	Basic	Basic	No	Yes	49
16	Yes	Yes	Basic	Basic	No	No	63
17	Yes	No	Enhanced	Enhanced	Yes	Yes	45
18	Yes	No	Enhanced	Enhanced	Yes	No	55
19	Yes	No	Enhanced	Enhanced	No	Yes	56
20	Yes	No	Enhanced	Enhanced	No	No	42
21	Yes	No	Enhanced	Basic	Yes	Yes	61
22	Yes	No	Enhanced	Basic	Yes	No	52
23	Yes	No	Enhanced	Basic	No	Yes	54
24	Yes	No	Enhanced	Basic	No	No	58
25	Yes	No	Basic	Enhanced	Yes	Yes	44
26	Yes	No	Basic	Enhanced	Yes	No	51
27	Yes	No	Basic	Enhanced	No	Yes	40
28	Yes	No	Basic	Enhanced	No	No	50
29	Yes	No	Basic	Basic	Yes	Yes	46
30	Yes	No	Basic	Basic	Yes	No	39
31	Yes	No	Basic	Basic	No	Yes	43
32	Yes	No	Basic	Basic	No	No	52

Each component had two levels: on vs. off, or enhanced vs. basic.

Participants answered demographic questions followed by a scenario asking them to imagine they had been diagnosed with breast cancer and had been prescribed AET (Supplementary Material 1). This scenario aimed to reflect the information received when women are prescribed AET, and received patient input. Participants could not proceed until 30 s had passed to encourage them to read the scenario. Participants then completed a questionnaire regarding their beliefs about AET, before being randomized to 1 of 32 experimental conditions. The relevant information leaflet was displayed, and they could not proceed until 3 min had passed. Following this, participants were asked to complete the same questionnaire about their beliefs about AET. Data were collected in May 2022.

#### Participants and setting

A market research company sent out the survey link to their panel of profiled respondents in the UK who have signed up to participate in market research. Participants confirmed they were female, over 18 and could read English. The market research company provided participants with a small incentive. The experiment took place online. We used a sample of healthy women as a pragmatic decision based on recruitment costs. This reflects the resource management principle in the MOST framework, which emphasizes the importance of making the best use of available resources through balancing cost and scientific yield [38].

#### Candidate intervention components

##### Constant component

This information was not empirically examined, as all participants received this component. It consisted of a title page, a description of the types of AET, an explanation about how AET works, and how to take AET.

##### Diagrams detailing the mechanisms of AET (*diagrams*)

Better understanding of how AET works has been associated with fewer concerns about AET [19]. Visual information, in the form of medical diagrams, may aid understanding as to how a medication works and can be easier to remember [39–41]. This component consisted of two levels; on, in which medical diagrams supplemented text explaining how AET works, and off, in which text alone explained the mechanisms of AET.

##### Information about the benefits of AET (*benefits*)

Beliefs about treatment control have correlated negatively with medication concerns, and positively with necessity beliefs [19]. Visual aids, such as icon arrays, can help readers understand information, and are helpful for those with low numeracy [42]. In the enhanced level, information was provided regarding the benefits of AET, with two icon arrays to support this. In the basic level, one statement acknowledged that AET reduced the risk of recurrence and mortality.

##### Information about the prevalence of side effects (*side effects*)

Misattributing symptoms to AET contributes to the nocebo effect, which can influence the formation of medication beliefs [31, 43–45]. Displaying frequencies of side effects using numerical values, positively framing side effect information (e.g., 99% of people will not experience this side effect), and informing people about the nocebo effect could lead to reduced attribution of symptoms to a medication [43, 46–48]. The enhanced level details the prevalence of side effects of AET, using positive framing. Additional text challenges attribution of side effects to the medication. The basic level includes a side effect table indicating which side effects are possible, but no information about their prevalence or attribution.

##### Answers to common concerns about AET (*concerns*)

Negative expectations about a medication contribute to the nocebo effect, and have been associated with increased side effect reporting in women taking AET [32, 44, 45]. Addressing common concerns could reduce negative expectations of AET. This component is made up of answers to four common concerns informed by existing qualitative studies and suggestions from our patient group [14–16]. For example, worry about not being able to cope with side effects was addressed by suggesting that for many women side effects are manageable, but that further support can be sought if they are disruptive. This component was either present or absent.

##### Input from breast cancer survivors (*patient input*)

Narrative information, such as patient stories, can increase engagement with educational materials [49]. This component comprises four quotes, photos from women with experience taking AET, and a statement highlighting the leaflet has been codesigned. This component was present or absent.

#### Measures

##### Participant characteristics

Information was collected regarding participant’s age, marital status, education level, ethnicity, menopausal status, and previous breast cancer diagnoses. If participants reported a breast cancer diagnosis, they were asked the stage and whether they had ever taken AET. All participants were additionally asked whether any close relations had been diagnosed with breast cancer.

##### Beliefs about Medication Questionnaire-AET (BMQ-AET)

The 10-item BMQ-AET was used to assess specific medication beliefs [50]. Participants responded on a 5-point scale ranging from “strongly disagree” to “strongly agree.” The BMQ-AET consists of two subscales; specific concerns and necessity beliefs, with five items relating to each subscale. As suggested by the authors of the original BMQ [17], and to reflect the need for a singular outcome capturing both necessity beliefs and concerns for a factorial experiment, we decided a priori to calculate a BMQ-AET differential score. This was calculated by subtracting concern from necessity scores (range −20 to +20).

#### Statistical considerations

##### Sample size

Sample size was calculated using the “MOST” package in R Studio [51]. To detect an effect size of 0.15, with 0.9 power and alpha set to 0.1, a sample size of 1,524 was required. It was assumed that 5% of participants would enter “nonsense” responses (defined as completing the survey in less than a third of the median time taken to complete the survey). Therefore, the sample size was increased to 1,604. The effect size chosen was based on the minimum effect of interest. Alpha was set to 0.1 rather than the traditional 0.05. This is due to the aim of this study being to screen components; incorrectly screening out and incorrectly screening in a component (the result of Type I and II error rates) are equally detrimental. This reflects the decision priority perspective taken in the optimization phase of MOST [52].

##### Randomization

Simple randomization was used in which each participant was randomly assigned to one of 32 experimental conditions [53]. The randomization was conducted automatically in the online survey platform, Qualtrics.

##### Missing data

Data for participants who did not complete the survey was not recorded. All fields in the survey were mandatory and therefore there was no missing data.

#### Statistical analysis

##### Primary analyses

The primary outcome was the BMQ-AET differential score after viewing the information leaflet. Descriptive statistics were used to summarize necessity belief, concern, and BMQ-AET differential scores overall and by component. Multiple linear regression with effect coding (−1, +1) was used to directly assess the main effects and the interaction effects of the components on the BMQ-AET differential. The model included all main effects and all interactions, and baseline BMQ-AET differential scores and age as covariates. Coefficients are reported as they originate from the model, which is half what they would traditionally be defined to be, due to the use of effect coding. Data were analyzed using R Statistical Software (R version 4.2.0, April 22, 2022) [54] on an intent-to-treat basis (R packages detailed in Supplementary Material 2).

##### Sensitivity analyses

We repeated the primary analysis removing speed responders, defined as anyone who fit one of three criteria: (i) completed the whole survey in less than a third of the median time it took participants to complete the survey, (ii) answered the same response to all items in the BMQ-AET pretest, and (iii) answered the same response to all items in the BMQ-AET posttest. Our second sensitivity analysis removed participants who reported a diagnosis of breast cancer, to assess if decisions would change without this group. Sensitivity analysis was not conducted for only participants reporting a breast cancer diagnosis due to the low number of participants (*n* = 79).

##### Screening decisions

A decision priority perspective was taken to select components to include in the optimized information leaflet [52]. The all-active components criterion was used to make screening decisions, which is defined as the best expected outcome irrespective of cost or other constraints [52]. The criteria for a component to be considered for inclusion in the optimized package was set a priori at *p* < .1 for main effects and interaction effects. Any main effects and interaction effects which were considered important (i.e., *p* < .1) were added into the parsimonious prediction model. Coefficients for all other effects not considered important (i.e., *p* > .1) were set to zero.

Decision-making followed a stepped approach [52]. Following the principle of “effect hierarchy,” which suggests that main effects and lower-order interaction effects are the most scientifically important, main effects were considered initially to screen components in and out [55]. Decisions were reconsidered in light of interaction effects, prioritizing lower-order interactions and those containing a component where a main effect was present. After considering all interactions, any components on the screened-in list were set to the higher level, and any components on the screened-out list were set to the lower level to make up the optimized information leaflet.

## Results

### Participant Characteristics

A total of 1,604 participants were randomized and completed the survey. One participant was removed due to being under 18 years old (Condition 29), leaving a primary population of 1,603 participants (Table 2). Most women were White British (88.8%), either married or living with a partner (61.9%), and around a third (34.1%) reported degree-level education. Seventy-nine (4.9%) women had a diagnosis of breast cancer, with 67/79 (84.8%) being estrogen or progesterone receptor positive. Fifty-eight women were currently taking AET or had previously taken AET. Table 3 displays the mean beliefs about medicines scores overall and by factor.

**Table 2.** Demographics of participants

Demographics	Total sample (*N* = 1,603)
Age, mean (SD, range)	47.93 (16.29; 18–83)
Marital status (%)	
Single	398 (24.8)
Married	749 (46.7)
Cohabiting/ living with a partner	244 (15.2)
Divorced/ separated	159 (9.9)
Widowed	53 (3.3)
Education (%)	
GCSE/O-Level/CSE	374 (23.3)
Vocational qualifications (NVQ1 + 2)	142 (8.9)
A-Level	269 (16.8)
Higher educational qualifications (below degree)	190 (11.9)
Degree-level education	547 (34.1)
Still studying	9 (0.6)
Other	18 (1.1)
No formal qualifications	54 (3.4)
Ethnicity (%)	
Asian or Asian British	78 (4.9)
Black or Black British (African)	16 (1.0)
Black or Black British (Caribbean)	10 (0.6)
Mixed	27 (1.7)
Chinese	6 (0.4)
White British	1,424 (88.8)
Other	36 (2.3)
Do not wish to answer	6 (0.4)
Menopausal status (%)	
Premenopausal	697 (43.5)
Postmenopausal	684 (42.7)
Unsure	222 (13.9)
Previous breast cancer diagnosis (%)	79 (4.9)
Stage of breast cancer (%)^a^	
Stage 0	3 (3.8)
Stage 1	25 (31.7)
Stage 2	22 (27.8)
Stage 3	11 (13.9)
Stage 4	1 (1.3)
Unsure	17 (21.5)
ER+ Breast cancer (%)^a^	
Yes	67 (84.8)
No	12 (15.2)
Experience with AET^a^	
Currently taking	35 (44.3)
Previously taken	23 (29.1)
No experience	15 (19.0)
Unsure	6 (7.6)
Type of hormone therapy^a^	
Tamoxifen	29 (36.7)
Anastrozole	22 (27.8)
Letrozole	17 (21.5)
Exemestane	3 (3.8)
Other	1 (1.3)
Close relations experience of breast cancer	732 (45.7)
Parent	167 (10.4)
Sibling	72 (4.5)
Grandparent	114 (7.1)
Partner	15 (0.9)
Close friend	311 (19.4)
Other	143 (8.9)

^a^Percentages calculated only from those who have had breast cancer (*n* = 79).

**Table 3.** Descriptives for baseline and follow-up beliefs about medicines scale scores (*n* = 1,603)

Factor level	Baseline, mean (SD)			Follow-up, mean (SD)		
	Necessity^a^	Concerns^a^	Differential^b^	Necessity^a^	Concerns^a^	Differential^b^
Total	17.99 (4.28)	16.47 (3.97)	1.52 (5.36)	18.73 (4.20)	16.43 (4.11)	2.31 (5.72)
Diagrams						
On	17.98 (4.36)	16.44 (4.03)	1.54 (5.46)	18.80 (4.27)	16.42 (4.16)	2.37 (5.93)
Off	18.00 (4.19)	16.50 (3.90)	1.50 (5.25)	18.67 (4.14)	16.43 (4.06)	2.24 (5.49)
Benefits						
On	17.99 (4.37)	16.60 (3.93)	1.39 (5.21)	18.70 (4.16)	16.54 (4.05)	2.16 (5.60)
Off	17.99 (4.18)	16.33 (4.00)	1.67 (5.52)	18.78 (4.25)	16.31 (4.17)	2.47 (5.84)
Side effects						
On	17.94 (4.31)	16.55 (3.94)	1.39 (5.25)	18.75 (4.21)	16.53 (4.00)	2.22 (5.51)
Off	18.04 (4.25)	16.39 (4.00)	1.64 (5.46)	18.71 (4.20)	16.33 (4.21)	2.38 (5.91)
Concerns						
On	17.88 (4.38)	16.34 (4.01)	1.54 (5.23)	18.60 (4.26)	16.27 (4.10)	2.33 (5.47)
Off	18.10 (4.17)	16.60 (3.92)	1.50 (5.49)	18.87 (4.14)	16.59 (4.11)	2.28 (5.96)
Patient input						
On	18.09 (4.27)	16.51 (3.95)	1.59 (5.44)	18.94 (4.26)	16.22 (4.08)	2.72 (5.74)
Off	17.89 (4.28)	16.43 (3.98)	1.46 (5.29)	18.54 (4.14)	16.63 (4.13)	1.91 (5.67)

^a^Possible range: 5–25.

^b^Possible range: −20 to +20.

### Engagement

The median time to complete the survey was 9.45 min (range = 4.87–85.25 min). The median time spent looking at the information leaflet (including the compulsory 3 min) ranged from 3.10 min (range = 3.02–29.28 min) in Condition 16, to 3.58 min in Condition 12 (range = 3.02–37.67 min) (Supplementary Material 3).

### Optimization Experiment

**Fig. 2.**
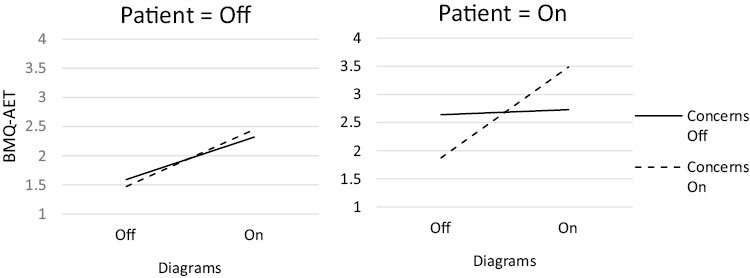
Three-way synergistic interaction between *patient input*, *diagrams*, and *concerns* components

**Fig. 3.**
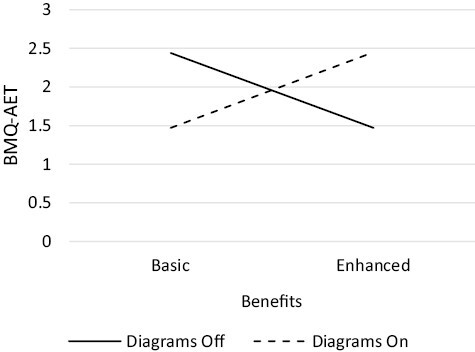
Two-way synergistic interaction between *benefits* and *diagrams* components

**Fig. 4.**
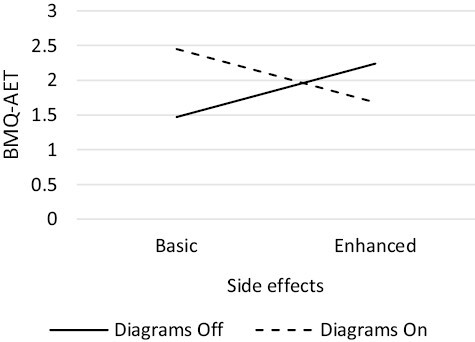
Two-way antagonistic interaction between *diagrams* and *side effects* components

**Fig. 5.**
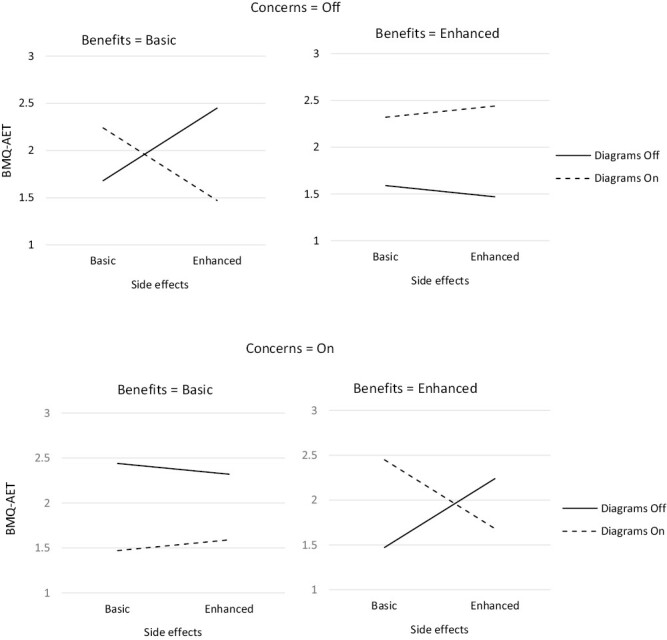
Four-way antagonistic interaction between *benefits*, *diagrams*, *concerns*, and *side effects* components.

The number of participants randomized to each of the 32 conditions ranged from 38 to 63 (Table 1). One component, *patient input,* had a statistically significant positive main effect on beliefs about AET (β = 0.063, 90% CI 0.035, 0.091, *p* < .001) (Table 4). There was one significant synergistic two-way interaction: *diagrams × benefits* (β = 0.047, 90% CI 0.019, 0.075, *p* = .006), in which the effect of *diagrams* was greater when *benefits* was enhanced. There was an antagonistic two-way interaction: *diagrams* × *side effects* (β = −0.029, 90% CI −0.056, −0.001, *p* = .093), in which the effect of *diagrams* was reduced when *side effects* was enhanced. There was a synergistic three-way interaction: *diagrams × concerns* × *patient input* (β = 0.029, 90% CI 0.001, 0.057, *p* = .085), in which the presence of all three components set to on/enhanced was greater than would be expected from each component alone. Finally, there was an antagonistic four-way interaction: *diagrams × benefits × side effects* × *concerns* (β = −0.038, 90% CI −0.066, −0.010, *p* = .024), in which *side effects* being enhanced reduced the effect of *diagrams, benefits, and concerns* (Figs. 2–5).

**Table 4.** Multiple linear regression showing the effect of candidate components on beliefs about AET

		Full regression model				Parsimonious prediction model			
		b-weight	β (90% CI)	*t*	*p*	b-weight	β (90% CI)	*t*	*p*
	Intercept	2.322		23.989	**<.001**	2.319		24.219	**<.001**
Main effects	Diagrams (D)	0.028	0.005 (−0.023, 0.033)	0.293	.770				
	Benefits (B)	−0.047	−0.008 (−0.036, 0.020)	−0.486	.627				
	Side effects (SE)	0.018	0.003 (−0.025, 0.031)	0.185	.853				
	Concerns (C)	−0.005	<0.001 (−0.029, 0.027)	−0.055	.956				
	Patient input (P)	0.362	0.063 (0.035, 0.091)	3.740	**<.001**	0.361	0.063 (0.036, 0.091)	3.773	**<.001**
Interactions	D × B	0.267	0.047 (0.019, 0.075)	2.757	**.006**	0.266	0.047 (0.019, 0.074)	2.770	**.006**
	D × SE	−0.163	−0.029 (−0.056, −0.001)	−1.683	**.093**	−0.163	−0.028 (−0.056, −0.001)	−1.693	**.091**
	B × SE	−0.102	−0.018 (−0.046, 0.010)	−1.051	.293				
	D × C	0.031	0.005 (−0.022, 0.033)	0.324	.746				
	B × C	−0.080	−0.014 (−0.042, 0.014)	−0.826	.409				
	SE × C	−0.072	−0.013 (−0.040, 0.015)	−0.745	.456				
	D × P	0.134	0.023 (−0.005, 0.051)	1.380	.168				
	B × P	0.002	<0.001 (−0.028, 0.028)	0.022	.983				
	SE × P	−0.121	−0.021 (−0.049, 0.007)	−1.253	.210				
	C × P	−0.035	−0.006 (−0.034, 0.022)	−0.357	.721				
	D × B × SE	−0.045	−0.008 (−0.036, 0.020)	−0.462	.644				
	D × B × C	−0.042	−0.007 (−0.035, 0.021)	−0.437	.663				
	D × SE × C	0.144	0.025 (−0.003, 0.053)	1.484	.138				
	B × SE × C	0.032	0.006 (−0.022, 0.033)	0.327	.744				
	D × B × P	0.086	0.015 (−0.013, 0.043)	0.888	.375				
	D × SE × P	0.130	0.023 (−0.005, 0.051)	1.344	.179				
	B × SE × P	0.061	0.011 (−0.017, 0.039)	0.632	.527				
	D × C × P	0.167	0.029 (0.001, 0.057)	1.726	**.085**	0.160	0.028 (0.000, 0.056)	1.664	**.096**
	B × C × P	0.047	0.008 (−0.020, 0.036)	0.481	.630				
	SE × C × P	−0.002	<0.001 (−0.028, 0.027)	−0.025	.980				
	D × B × SE × C	−0.219	−0.038 (−0.066, −0.010)	−2.261	**.024**	−0.224	−0.039 (−0.067, −0.012)	−2.332	**.020**
	D × B × SE × P	−0.096	−0.017 (−0.045, 0.011)	−0.987	.324				
	D × B × C × P	−0.157	−0.027 (−0.055, 0.001)	−1.614	.107				
	D × SE × C × P	0.070	0.012 (−0.016, 0.040)	0.724	.469				
	B × SE × C × P	0.107	0.019 (−0.009, 0.047)	1.105	.269				
	D × B × SE × C × P	0.095	0.017 (−0.011, 0.045)	0.980	.327				
Covariates	Baseline BMQ-AET	0.784	0.735 (0.707, 0.763)	42.842	**<.001**	0.785	0.736 (0.708, 0.764)	43.291	**<.001**
	Age	0.003	0.010 (−0.018, 0.038)	0.575	.566	0.005	0.014 (−0.014, 0.042)	0.846	.397

Based on this analysis, we constructed the parsimonious prediction model, containing only main effects and interactions meeting the threshold for importance (*p* < .1). Due to imbalance in the number of participants across conditions, the analysis was repeated including only the main effects and interactions of importance, and the covariates, baseline BMQ-AET and age [52]. There was minimal change in the coefficient values (Table 4).

### Decision-making

Initially, the only component with an important main effect, *patient input,* was screened in. We then reconsidered the screened in and out lists based on the important interaction effects (*p* < .1). We examined the three-way *diagrams × concerns* × *patient input* interaction first, as this contained a component with a main effect (*patient input*). When *patient input* was set to on, the effect of *concerns* was higher when *diagrams* was also set to on. Setting all three components to the higher levels had the optimum effect (Fig. 2). Therefore, *concerns* and *diagrams* were screened in.

Next, we examined the *diagrams* × *benefits* interaction (Fig. 3). There was a significant synergistic interaction in which the effect of *diagrams* was increased when *benefits* was set to on. The optimum effect occurred when either both components were set to the higher or lower level. As *diagrams* was screened in previously, it was more beneficial to screen in *benefits*, rather than screen out both *benefits* and *diagrams*.

The antagonistic *diagrams × side effects* interaction highlights the effect of *diagrams* was reduced when *side effects* was set to the higher level (Fig. 4). When both components were set to the higher level, the BMQ-AET differential was smaller than would be expected with no interaction. Therefore, *side effects* remained screened out.

Finally, we examined the four-way *diagrams × benefits × side effects* × *concerns* interaction (Fig. 5). Here we examined what effect *side effects* would have when all other components involved are set to the higher levels, as this reflected the screened-in and screened-out list at this stage. When *diagrams, benefits,* and *concerns* were set to their higher levels, *side effects* being set to the higher level diminished the effect. Therefore, *side effects* remained screened out, meaning the basic level of *side effects* was included in the optimized information leaflet.

Table 5 lists the predicted outcomes for *Ŷ*_Beliefs_ for all 16 conditions reflecting all combinations of the four screened-in components, computed using the expression for the parsimonious prediction model. Condition 5 had the greatest *Ŷ*_Beliefs_ value, which represents *diagrams, benefits, concerns,* and *patient input* being screened in, and *side effects* screened out.

**Table 5.** Predicted beliefs about medications scores for each condition

Condition	Side effects	Diagrams	Benefits	Concerns	Patient input	*Ŷ*_Beliefs_ ^a^	*Ŷ*_Beliefs_ ^b^
**5**	**Basic**	**On**	**Enhanced**	**On**	**On**	**2.524**	**4.315**
6	Basic	On	Enhanced	On	Off	2.342	4.133
7	Basic	On	Enhanced	Off	On	2.390	4.181
8	Basic	On	Enhanced	Off	Off	2.320	4.111
13	Basic	On	Low	On	On	2.352	4.143
14	Basic	On	Low	On	Off	2.170	3.961
15	Basic	On	Low	Off	On	2.374	4.165
16	Basic	On	Low	Off	Off	2.304	4.095
21	Basic	Off	Enhanced	On	On	2.240	4.031
22	Basic	Off	Enhanced	On	Off	2.170	3.961
23	Basic	Off	Enhanced	Off	On	2.374	4.165
24	Basic	Off	Enhanced	Off	Off	2.192	3.983
29	Basic	Off	Low	On	On	2.412	4.203
30	Basic	Off	Low	On	Off	2.342	4.133
31	Basic	Off	Low	Off	On	2.390	4.181
32	Basic	Off	Low	Off	Off	2.208	3.999

^a^Predicted values calculated for the parsimonious model without covariates.

^b^Predicted values calculated for the parsimonious model with covariates.

### Sensitivity Analyses

When removing speed responders (*n* = 153), the results were consistent with the primary analysis (Supplementary Material 4). The only important effect to change was the three-way *diagrams × concerns × patient input* interaction which became nonsignificant (*p* = .103), but this did not impact which components were screened out. Demographic and clinical characteristics were comparable between women with and without breast cancer (Supplementary Material 4). There was no significant difference in baseline BMQ-AET differential scores between women with breast cancer (*M* = 2.19, *SD* = 5.93) and women without breast cancer (*M* = 1.49, *SD* = 5.33) *t*(1,601) = 1.14, *p = .259*. Women with breast cancer had significantly higher baseline necessity beliefs (*M* = 18.92, *SD* = 4.27) than those without breast cancer (*M* = 17.94, *SD* = 4.27), *t*(1,601) = 1.99, *p* = .047 (Supplementary Material 4). When removing participants reporting a diagnosis of breast cancer (*n* = 79), results were consistent with the primary analysis and decision-making did not change (Supplementary Material 4).

## Discussion

Using an online factorial screening experiment, we optimized an information leaflet intervention to increase beliefs about the necessity of AET and reduce concerns about AET. The optimized information leaflet contained four out of five of the candidate components; diagrams explaining how AET works *(diagrams)*, icon arrays explaining the benefits of AET *(benefits)*, answers to common concerns about AET *(concerns),* and quotes and photographs of breast cancer survivors explaining their motivations for taking AET *(patient input)*. The side effect component *(side effects)* was screened out due to interacting negatively with the other candidate components. The optimization process led to development of a more efficient and effective information leaflet.

We have demonstrated that it is feasible and beneficial to optimize an information leaflet using an online factorial experiment. Compared with a classical approach (i.e., using an RCT to evaluate the leaflet as a package), the optimization phase provided an insight into the contributions of individual components of the leaflet in isolation and combined. From this, we know that the leaflet supports medication beliefs, which is a known barrier to AET adherence [6, 10–16]. The resulting leaflet is optimized to increase efficiency (e.g., redundant components are not included) and effectiveness (e.g., only components reaching an a priori statistical significance are included).

The strategies we tested appear to be effective in changing medication beliefs, which builds on the limited existing evidence. These strategies could be applied in other contexts where medication beliefs are a barrier to adherence behaviors. However, our results suggest these strategies had more impact on increasing necessity beliefs than reducing concerns. While this was still effective in improving the cost-benefit analysis (differential) which has been found to be a more consistent predictor of nonadherence than necessity beliefs or concerns alone [56], future research could focus on developing components to better reduce concerns.

The *patient input* component was the only candidate component to demonstrate a main effect on beliefs about AET. In our conceptual model, we hypothesized that this component would interact with all other components, but it did not interact with the *side effects* and *benefits* components. The main effect suggests that the *patientinput* component has an alternative mechanism for affecting beliefs about AET. One explanation is that the content of the quotes could have led to social comparison; in which participants may have adapted their beliefs after comparing with others, which is common in a state of uncertainty [57, 58]. Information about the main effects and interaction effects obtained in an optimization experiment enables refinement of our conceptual model and understanding of how interventions may work.

The only candidate component screened out of the optimized information leaflet was the *sideeffects* component. Informing participants of the nocebo effect (suggesting that not all physiological sensations may be caused by AET), and providing positively framed side effect information did not affect medication beliefs, and interacted negatively with the *diagrams, benefits* and *concerns* components. The lower level of this component could have provided the “gist” of the information sufficiently (i.e., the bottom line meaning that different side effects are possible for different types of AET). According to Fuzzy Trace Theory, health information may be encoded in two ways; a gist representation (the essence of the information), and a verbatim representation (literal, precise information, e.g., specific statistics) [59]. When making decisions, people tend to prefer to rely on the gist representation [59, 60]. In this case, the lower level of the *sideeffect* component may have been enough to form this gist-based representation, meaning the enhanced level of the component was redundant. Alternatively, participants may not have understood the enhanced side effect information, or a written intervention may not be sufficient to reduce concerns. Screening out the enhanced *sideeffect* component led to a more efficient information leaflet, with redundant information removed. Future work could explore alternative methods to reduce concerns further.

The synergistic interaction between the *diagrams* and *benefits* components was the only hypothesized interaction evident in our data. The lack of main effect but the presence of a synergistic interaction indicates these components only work together. Understanding how a medication works via the *diagrams* component may increase understanding and belief in the benefits of AET [61]. Therefore, it may be appropriate to combine these components into a single, more robust component [52].

Our study had limitations. Women with breast cancer reported significantly higher necessity beliefs at baseline than women without breast cancer (Supplementary Material 4), which could limit the generalizability of the findings to women with breast cancer. However, the concerns and differential scores were not significantly different between women with and without breast cancer at baseline or follow-up (Supplementary Material 4). BMQ-AET scores for the total sample and breast cancer subsample were comparable to previous published studies conducted with women with breast cancer [34, 62]. Further evaluation of the leaflet will be conducted in women with breast cancer. The majority of participants were White British and had higher level educational qualifications. A more diverse sample may have generated different findings that reflected a different optimal combination of components. As a result of using simple randomization, the number of participants in each experimental condition was not balanced which will have reduced statistical power. We optimized an information leaflet based on one singular outcome, but other outcomes could also be considered, such as women’s satisfaction with the information they receive. Further work is needed to explore optimization with multiple outcomes of interest. To limit the length of the survey, we did not include assessments of each component target (e.g., coherence). Future optimization studies could include these assessments to enable causal pathway analyses to enhance our understanding of the underlying mechanisms of action [63].

We used a rigorous approach to optimize an information leaflet to increase necessity beliefs and reduce concerns in women taking AET. Our approach has enabled refinement of our conceptual model, and has led to the development of a more efficient information leaflet, removing components that are negatively impacting the outcome. Factorial experimental designs offer a highly efficient way of optimizing multicomponent intervention packages such as information leaflets. Optimization, guided by MOST, can enhance our overall understanding of behavioral interventions.

## Supplementary Material

kaad037_suppl_Supplementary_Material_1

kaad037_suppl_Supplementary_Material_2

kaad037_suppl_Supplementary_Material_3

kaad037_suppl_Supplementary_Material_4
