# Supplementary material for: Optimization of an Information Leaflet to Influence Medication Beliefs in Women With Breast Cancer: A Randomized Factorial Experiment
Source: Ann Behav Med. 2023 Jul 26;57(11):988–1000. doi: 10.1093/abm/kaad037 (PMC10578395; doi:10.1093/abm/kaad037)
Supplement: kaad037_suppl_Supplementary_Material_1 [file kaad037_suppl_supplementary_material_1.docx]

**Contextual Scenario**

All participants were presented with the following text prior to completing the baseline questionnaire. Participants were unable to move to the next page until 30 seconds had passed.

Some of you may have been diagnosed with breast cancer and may have had experience with adjuvant hormone therapy, a commonly prescribed treatment for breast cancer. Others will not have had experience of breast cancer, or these medications specifically. If you have not had experience of breast cancer and hormone therapy, please read the following scenario prior to beginning this survey. This explains the context in which hormone therapy would be prescribed as part of treatment for breast cancer. Please imagine you have been prescribed hormone therapy for breast cancer for the remainder of the survey.

*Imagine you have received a diagnosis of oestrogen receptor-positive breast cancer, which is  a specific type of breast cancer. The breast cancer has been found early which means it can be treated with the aim of curing it.*

*You have had surgery to remove the cancerous tumour, and have received radiotherapy and/or chemotherapy to get rid of any cancer cells that might have been left behind. This aims to reduce the chance that the cancer will return.*

*In the final appointment with your oncologist (the doctor that is coordinating  your treatment plan), you have been told you will be prescribed a hormone therapy, and that you must take this medication (a small tablet) every day for the next 5 years, which could be increased to 10 years. You are told that this medication can reduce your risk of the breast cancer returning. You do not know much about this medication, but have heard from other women that it may cause some uncomfortable side effects like hot flushes or joint pain. You are then discharged from the hospital, and are told to reorder your prescription via your GP. It is possible that you will experience some side effects if you take the hormone therapy, but taking the hormone therapy can also reduce the chance that the cancer will come back.*

Please note, you will only be able to proceed to the next page once 30 seconds has passed.
