# Supplementary material for: Optimization of an Information Leaflet to Influence Medication Beliefs in Women With Breast Cancer: A Randomized Factorial Experiment
Source: Ann Behav Med. 2023 Jul 26;57(11):988–1000. doi: 10.1093/abm/kaad037 (PMC10578395; doi:10.1093/abm/kaad037)
Supplement: kaad037_suppl_Supplementary_Material_2 [file kaad037_suppl_supplementary_material_2.docx]

**R Packages Used for Analysis**

The following R packages were used;

1. dplyr (v1.0.10) (1)
2. summarytools (v1.0.1) (2)
3. parameters (v0.18.2) (3)
4. car (v3.1.0) (4)
5. sjPlot (v2.8.12) (5)
6. ggeffects (v1.1.3) (6).

1. Wickham H FR, Henry L, Müller K: _dplyr: A Grammar of Data Manipulation_. R package version 1.0.10. 2022.

2. Comtois D: _summarytools: Tools to Quickly and Neatly Summarize Data_. R package version 1.0.1. 2022.

3. Lüdecke D B-SM, Patil I, Makowski D,: Extracting, Computing and Exploring the Parameters of Statistical Models using R. *Journal of Open Source Software.* 2020, *5:*2445.

4. Fox J WS: An {R} Companion to Applied Regression. (Vol. 3rd). Thousand Oaks CA: Sage, 2019.

5. Lüdecke D: sjPlot: Data Visualization for Statistics in Social Science_. R package version 2.8.12. 2022.

6. Lüdecke D: ggeffects: Tidy Data Frames of Marginal Effects from Regression Models. *Journal of Open Source Software*, *3:*772.
