# Supplementary material for: Optimization of an Information Leaflet to Influence Medication Beliefs in Women With Breast Cancer: A Randomized Factorial Experiment
Source: Ann Behav Med. 2023 Jul 26;57(11):988–1000. doi: 10.1093/abm/kaad037 (PMC10578395; doi:10.1093/abm/kaad037)
Supplement: kaad037_suppl_Supplementary_Material_3 [file kaad037_suppl_supplementary_material_3.docx]

| **Table 1**  *Engagement with information leaflet* | |
| --- | --- |
| Experimental Condition | Median time spent on information leaflet page (Range), in minutes |
| Condition 1 | 3.22 (3.0-7.93) |
| Condition 2 | 3.20 (3.02-10.42) |
| Condition 3 | 3.32 (3.02-7.80) |
| Condition 4 | 3.35 (3.02-10.33) |
| Condition 5 | 3.23 (3.02- 10.87) |
| Condition 6 | 3.12 (3.02- 10.18) |
| Condition 7 | 3.52 (3.02- 12.25) |
| Condition 8 | 3.40 (3.02- 11.47) |
| Condition 9 | 3.15 (3.02- 13.93) |
| Condition 10 | 3.33 (3.02- 42.48) |
| Condition 11 | 3.18 (3.03- 8.08) |
| Condition 12 | 3.58 (3.02- 37.37) |
| Condition 13 | 3.22 (3.02- 7.18) |
| Condition 14 | 3.23 (3.02- 10.27) |
| Condition 15 | 3.18 (3.00- 7.20) |
| Condition 16 | 3.10 (3.02- 29.28) |
| Condition 17 | 3.18 (3.02- 6.73) |
| Condition 18 | 3.28 (3.02- 11.30) |
| Condition 19 | 3.43 (3.02- 9.32) |
| Condition 20 | 3.43 (3.02- 9.32) |
| Condition 21 | 3.27 (3.02- 15.05) |
| Condition 22 | 3.40 (3.03- 16.80) |
| Condition 23 | 3.35 (3.02- 9.40) |
| Condition 24 | 3.33 (3.02- 10.88) |
| Condition 25 | 3.33 (3.03- 10.15) |
| Condition 26 | 3.22 (3.02- 13.05) |
| Condition 27 | 3.37 (3.03- 10.93) |
| Condition 28 | 3.33 (3.02- 16.52) |
| Condition 29 | 3.18 (3.02- 6.18) |
| Condition 30 | 3.20 (3.02- 8.58) |
| Condition 31 | 3.12 (3.02- 6.33) |
| Condition 32 | 3.17 (3.02- 8.25) |

**Engagement with the Information Leaflet**
