# Supplementary material for: Optimization of an Information Leaflet to Influence Medication Beliefs in Women With Breast Cancer: A Randomized Factorial Experiment
Source: Ann Behav Med. 2023 Jul 26;57(11):988–1000. doi: 10.1093/abm/kaad037 (PMC10578395; doi:10.1093/abm/kaad037)
Supplement: kaad037_suppl_Supplementary_Material_4 [file kaad037_suppl_supplementary_material_4.docx]

| **Table 1**  *Sensitivity regression analysis of the primary analysis removing speed responders* | | | | | |
| --- | --- | --- | --- | --- | --- |
|  |  | **Beta** | **β (90% CI)** | **t** | ***p*** |
|  | Intercept | 2.405 |  | 23.023 | **<0.001** |
| Main effects | Diagrams (D) | 0.063 | 0.011 (-0.018, 0.040) | 0.601 | 0.548 |
|  | Benefits (B) | -0.019 | -0.003 (-0.032, 0.026) | -0.182 | 0.856 |
|  | Side effects (SE) | -0.014 | -0.002 (-0.031, 0.027) | -0.138 | 0.890 |
|  | Concerns (C) | -0.036 | -0.006 (-0.035, 0.023) | -0.346 | 0.729 |
|  | Patient (P) | 0.402 | 0.068 (0.039, 0.097) | 3.852 | **<0.001** |
| Interactions | D x B | 0.261 | 0.044 (0.015, 0.073) | 2.495 | **0.013** |
|  | D x SE | -0.183 | -0.031 (-0.060, -0.002) | -1.755 | **0.079** |
|  | B x SE | -0.097 | -0.016 (-0.045, 0.013) | -0.929 | 0.353 |
|  | D x C | 0.079 | 0.013 (-0.016, 0.042) | 0.759 | 0.448 |
|  | B x C | -0.044 | -0.007 (-0.036, 0.022) | -0.422 | 0.673 |
|  | SE x C | -0.094 | -0.016 (-0.045, 0.013) | -0.904 | 0.366 |
|  | D x P | 0.148 | 0.025 (-0.004, 0.054) | 1.415 | 0.157 |
|  | B x P | 0.026 | 0.004 (-0.025, 0.033) | 0.247 | 0.805 |
|  | SE x P | -0.129 | -0.022 (-0.051, 0.007) | -1.233 | 0.218 |
|  | C x P | -0.049 | -0.008 (-0.037, 0.021) | -0.465 | 0.642 |
|  | D x B x SE | -0.070 | -0.012 (-0.041, 0.017) | -0.670 | 0.503 |
|  | D x B x C | -0.037 | -0.006 (-0.035, 0.023) | -0.357 | 0.721 |
|  | D x SE x C | 0.120 | 0.020 (-0.009, 0.049) | 1.148 | 0.251 |
|  | B x SE x C | 0.032 | 0.005 (-0.024, 0.034) | 0.310 | 0.757 |
|  | D x B x P | 0.063 | 0.011 (-0.018, 0.040) | 0.608 | 0.543 |
|  | D x SE x P | 0.121 | 0.021 (-0.008, 0.050) | 1.164 | 0.244 |
|  | B x SE x P | 0.069 | 0.012 (-0.017, 0.041) | 0.660 | 0.510 |
|  | D x C x P | 0.170 | 0.029 (0.000, 0.058) | 1.632 | 0.103 |
|  | B x C x P | 0.002 | <0.001 (-0.029, 0.029) | 0.019 | 0.985 |
|  | SE x C x P | 0.006 | <0.001 (-0.028, 0.030) | 0.054 | 0.957 |
|  | D x B x SE x C | -0.197 | -0.033 (-0.062, -0.004) | -1.892 | **0.059** |
|  | D x B x SE x P | -0.082 | -0.014 (-0.043, 0.015) | -0.794 | 0.427 |
|  | D x B x C x P | -0.170 | -0.029 (-0.058, 0.000) | -1.624 | 0.105 |
|  | D x SE x C x P | 0.070 | 0.012 (-0.017, 0.041) | 0.670 | 0.503 |
|  | B x SE x C x P | 0.108 | 0.018 (-0.011, 0.047) | 1.029 | 0.304 |
|  | D x B x SE x C x P | 0.140 | 0.024 (-0.005, 0.053) | 1.338 | 0.181 |
| Covariates | Baseline BMQ | 0.792 | 0.744 (0.715, 0.773) | 41.686 | **<0.001** |
|  | Age | <0.001 | -0.002 (-0.032, 0.027) | -0.120 | 0.905 |
| *Note.* Bold text indicates statistical significance (*p*<0.1)  *N* = 1450  Speed responders were classified participants who completed the survey in less than a third of the median time taken, or who answered the same response across all pretest or posttest BMQ-AET questionnaires.  Key: BMQ = Beliefs about medicines questionnaire. | | | | | |

**Sensitivity Analyses**

| **Table 2**  *Demographics of participants split by presence of breast cancer diagnosis* | | | | | |  |
| --- | --- | --- | --- | --- | --- | --- |
| Demographics | | | Total sample (*n=1603)* | | Women reporting breast cancer diagnosis (*n*=79) | Women not reporting breast cancer diagnosis (*n*=1,524) |
| Age, mean(SD, range) | | | 47.93 (16.29, 18-83) | | 51.62 (17.36, 18-79) | 47.74 (16.22, 18-83) |
| Marital Status (%) | | |  | |  |  |
| Single | | | 398 (24.8) | | 10 (12.7) | 388 (25.5) |
| Married | | | 749 (46.7) | | 55 (69.6) | 694 (45.5) |
| Cohabiting/ living with a partner | | | 244 (15.2) | | 4 (5.1) | 240 (15.8) |
| Divorced/ separated | | | 159 (9.9) | | 9 (11.4) | 150 (9.8) |
| Widowed | | | 53 (3.3) | | 1 (1.3) | 52 (3.4) |
| Education (%) | | |  | |  |  |
| GCSE/O-Level/ CSE | | | 374 (23.3) | | 17 (21.5) | 357 (23.4) |
| Vocational Qualifications (NVQ1+2) | | | 142 (8.9) | | 8 (10.1) | 134 (8.8) |
| A-Level | | | 269 (16.8) | | 12 (15.2) | 257 (16.9) |
| Higher educational qualifications (below degree) | | | 190 (11.9) | | 9 (11.4) | 181 (11.9) |
| Degree level education | | | 547 (34.1) | | 24 (30.4) | 523 (34.3) |
| Still Studying | | | 9 (0.6) | | 0 (0.0) | 9 (0.6) |
| Other | | | 18 (1.1) | | 2 (2.5) | 16 (1.0) |
| No formal qualifications | | | 54 (3.4) | | 7 (8.9) | 47 (3.1) |
| Ethnicity (%) | | |  | |  |  |
| Asian or Asian British | | | 78 (4.9) | | 3 (3.8) | 75 (4.9) |
| Black or Black British (African) | | | 16 (1.0) | | 1 (1.3) | 15 (1.0) |
| Black or Black British (Caribbean) | | | 10 (0.6) | | 2 (2.5) | 8 (0.5) |
| Mixed | | | 27 (1.7) | | 2 (2.5) | 25 (1.6) |
| Chinese | | | 6 (0.4) | | 0 (0.0) | 6 (0.4) |
| White British | | | 1424 (88.8) | | 71 (89.9) | 1353 (88.8) |
| Other | | | 36 (2.3) | | 0 (0.0) | 36 (2.4) |
| Do not wish to answer | | | 6 (0.4) | | 0 (0.0) | 6 (0.4) |
| Menopausal status (%) | | |  | |  |  |
| Premenopausal | | | 697 (43.5) | | 31 (39.2) | 666 (43.7) |
| Postmenopausal | | | 684 (42.7) | | 45 (57.0) | 639 (41.9) |
| Unsure | | | 222 (13.9) | | 3 (3.8) | 219 (14.4) |
|  |  |  | |  |  |  |

| **Table 3**  *Comparison between baseline and follow-up BMQ scores between women with and without breast cancer* | | | | | | | | | | |
| --- | --- | --- | --- | --- | --- | --- | --- | --- | --- | --- |
|  | **Baseline** | | | | | **Follow-up** | | | | |
|  | **BC, mean (SD)** | **No BC, mean (SD)** | ***t* ( 95% CI)** | **df** | ***p*** | **BC, mean (SD)** | **No BC, mean (SD)** | ***t* (95% CI)** | **df** | ***p*** |
| Necessity^a^ | 18.92 (4.27) | 17.94 (4.27) | 1.99 (0.01, 1.95) | 1601 | **0.047** | 19.11 (4.23) | 18.72 (4.20) | 0.82 (-0.55, 1.35) | 1601 | 0.411 |
| Concerns^a^ | 16.73 (5.07) | 16.46 (3.90) | 0.48 (-0.87, 1.43) | 82.86* | 0.632 | 16.57 (4.95) | 16.42 (4.06) | 0.26 (-0.98, 1.27) | 83.55* | 0.794 |
| Differential^b^ | 2.19 (5.93) | 1.49 (5.33) | 1.14 (-0.51, 1.92) | 1601 | 0.259 | 2.54 (5.75) | 2.29 (5.72) | 0.38 (-1.04, 1.54) | 1601 | 0.704 |
| *Note.* ^a^Possible range: 5-25  ^b^Possible range: -20 to +20  BC = participants reporting a diagnosis of breast cancer, *n*=79  No BC = participants not reporting a diagnosis of breast cancer, *n*=1, 524  * Indicates equal variances not assumed  Bold text indicates statistical significance (*p*<0.05) | | | | | | | | | | |

| **Table 4**  *Sensitivity analysis of the primary analysis removing women with breast cancer n=1524.* | | | | | |
| --- | --- | --- | --- | --- | --- |
|  |  | **Beta** | **β (90% CI)** | **t** | ***p*** |
|  | Intercept | 2.338 |  | 23.318 | **<0.001** |
| Main effects | Diagrams (D) | 0.067 | 0.012 (-0.017, 0.041) | 0.672 | 0.502 |
|  | Benefits (B) | -0.066 | -0.011 (-0.040, 0.017) | -0.654 | 0.514 |
|  | Side effects (SE) | 0.064 | 0.011 (-0.018, 0.040) | 0.638 | 0.523 |
|  | Concerns (C) | -0.030 | -0.005 (-0.034, 0.024) | -0.303 | 0.762 |
|  | Patient (P) | 0.373 | 0.065 (0.036, 0.094) | 3.723 | **<0.001** |
| Interactions | D x B | 0.290 | 0.051 (0.022, 0.080) | 2.892 | **0.004** |
|  | D x SE | -0.171 | -0.030 (-0.059, -0.001) | -1.704 | **0.089** |
|  | B x SE | -0.115 | -0.020 (-0.049, 0.009) | -1.145 | 0.252 |
|  | D x C | 0.020 | 0.003 (-0.025, 0.032) | 0.196 | 0.845 |
|  | B x C | -0.075 | -0.013 (-0.042, 0.016) | -0.749 | 0.454 |
|  | SE x C | -0.091 | -0.016 (-0.045, 0.013) | -0.908 | 0.364 |
|  | D x P | 0.127 | 0.022 (-0.007, 0.051) | 1.268 | 0.205 |
|  | B x P | 0.015 | 0.003 (-0.026, 0.031) | 0.145 | 0.884 |
|  | SE x P | -0.124 | -0.022 (-0.051, 0.007) | -1.241 | 0.215 |
|  | C x P | -0.060 | -0.010 (-0.039, 0.018) | -0.595 | 0.552 |
|  | D x B x SE | -0.032 | -0.006 (-0.034, 0.023) | -0.318 | 0.751 |
|  | D x B x C | -0.069 | -0.012 (-0.041, 0.017) | -0.690 | 0.490 |
|  | D x SE x C | 0.128 | 0.022 (-0.006, 0.051) | 1.281 | 0.200 |
|  | B x SE x C | 0.038 | 0.007 (-0.022, 0.036) | 0.382 | 0.703 |
|  | D x B x P | 0.072 | 0.013 (-0.016, 0.042) | 0.720 | 0.471 |
|  | D x SE x P | 0.132 | 0.023 (-0.006, 0.052) | 1.316 | 0.189 |
|  | B x SE x P | 0.057 | 0.010 (-0.019, 0.039) | 0.566 | 0.571 |
|  | D x C x P | 0.196 | 0.034 (0.005, 0.063) | 1.955 | **0.051** |
|  | B x C x P | 0.502 | 0.009 (-0.020, 0.038) | 0.500 | 0.617 |
|  | SE x C x P | -0.001 | <0.001 (-0.029, 0.029) | -0.012 | 0.990 |
|  | D x B x SE x C | -0.209 | -0.037 (-0.065, -0.008) | -2.083 | **0.037** |
|  | D x B x SE x P | -0.088 | -0.015 (-0.044, 0.013) | -0.882 | 0.378 |
|  | D x B x C x P | -0.152 | -0.027 (-0.056, 0.002) | -1.508 | 0.132 |
|  | D x SE x C x P | 0.050 | 0.009 (-0.020, 0.038) | 0.502 | 0.616 |
|  | B x SE x C x P | 0.104 | 0.018 (-0.011, 0.047) | 1.038 | 0.299 |
|  | D x B x SE x C x P | 0.111 | 0.019 (-0.010, 0.048) | 1.101 | 0.271 |
| Covariates | Baseline BMQ | 0.783 | 0.730 (0.701, 0.759) | 41.228 | **<0.001** |
|  | Age | 0.004 | 0.011 (-0.019, 0.040) | 0.601 | 0.548 |
| *Note.* Bold text indicates statistical significance (p<0.1)  Anyone who answered yes to having breast cancer was removed from analysis (n=79)  Key: BMQ = Beliefs about medicines questionnaire. | | | | | |
